# Integrative subspace clustering by common and specific decomposition for applications on cancer subtype identification

**DOI:** 10.1186/s12920-019-0633-1

**Published:** 2019-12-24

**Authors:** Yin Guo, Huiran Li, Menglan Cai, Limin Li

**Affiliations:** School of Mathematics and Statistics, Xi’an Jiaotong University, Xianning West 28, Xi’an, China

**Keywords:** Subtype identification, Multi-view clustering, Subspace clustering

## Abstract

**Background:**

Recent high throughput technologies have been applied for collecting heterogeneous biomedical omics datasets. Computational analysis of the multi-omics datasets could potentially reveal deep insights for a given disease. Most existing clustering methods by multi-omics data assume strong consistency among different sources of datasets, and thus may lose efficacy when the consistency is relatively weak. Furthermore, they could not identify the conflicting parts for each view, which might be important in applications such as cancer subtype identification.

**Methods:**

In this work, we propose an integrative subspace clustering method (ISC) by common and specific decomposition to identify clustering structures with multi-omics datasets. The main idea of our ISC method is that the original representations for the samples in each view could be reconstructed by the concatenation of a common part and a view-specific part in orthogonal subspaces. The problem can be formulated as a matrix decomposition problem and solved efficiently by our proposed algorithm.

**Results:**

The experiments on simulation and text datasets show that our method outperforms other state-of-art methods. Our method is further evaluated by identifying cancer types using a colorectal dataset. We finally apply our method to cancer subtype identification for five cancers using TCGA datasets, and the survival analysis shows that the subtypes we found are significantly better than other compared methods.

**Conclusion:**

We conclude that our ISC model could not only discover the weak common information across views but also identify the view-specific information.

## Background

With the advancements of biological technologies, there are many kinds of data available such as genomic DNA copy number arrays, DNA methylation, exome sequencing, messenger RNA arrays, microRNA sequencing and reverse-phase protein arrays and so on. By analyzing the multiple data generated by cancer patients, it is now possible to classify cancer patients to different subgroups, and thus improve the diagnostic and treatment. For example, Breast cancer is one of the most common cancers worldwide, and it is clinically categorized into four basic therapeutic subgroups: (1). Luminal A with oestrogen receptor (ER) positive group; (2). Luminal B with oestrogen receptor (ER) positive group; (3) HER2 amplified group; (4) triple-negative breast cancers (TNBCs, also called basal-like, lacking expression of ER, progesterone receptor (PR) and HER2). The ER positive (including Luminal A and B) is the most common and diverse, and several genomic tests can be used to predict outcomes for ER+ patients receiving endocrine therapy. The treatment for the HER2 amplified subtype has a great success due to the effective therapeutic targeting of HER2. The basal-like breast cancers, often with BRCA1 mutations or of African ancestry have only option of chemotherapy. Therefore, subtype identification for breast cancers surely can assist the treatment for the patients.

Most molecular studies of subtype identification for breast cancer integrate genomic, epigenomic, and transcriptomic profiling including mRNA expression profiling, miRNA expression, DNA methylation and DNA copy number analysis, and so on. It is assumed in these studies that integrative clustering of multi-omics data can capture clearer structure that can not be discovered by only exploring a single omic data. In fact, in many other applications, a single object often can be represented by multiple features or views. For example, an image can be represented by its pixels and its captions, an Internet webpage can be represented by its text contents and the hyperlinks to other webpages, and a scientific publication can be represented by its text contents and its citations. In all these applications, multi-view clustering takes information from all views into account such that better clustering structures could be discovered.

The difficulty in multi-view learning mainly lies in that the similarity measurement, geometric distribution, clustering structure, and noisy levels and so on are often diverse for different views. Samples represented in different views may have their own clustering structures, or subspaces they lie in. The differences hamper the clustering significantly. It is challenging to efficiently reconcile the conflicting information among views.

Most of existing multi-view clustering approaches follow three directions. The first class of methods [1–7] attempt to determine new representations by minimizing the differences or maximizing the correlations between different views. The second class of approaches propagate information from different views to construct graphs or similarities in a slightly different way, including multi-view EM [8], multi-view spectral clustering [9, 10], multi-view clustering with unsupervised feature selection [11, 12], nonnegative Matrix Factorization [13], pattern fusion [14], similarity network fusion (SNF) [4]. For example, the similarity network fusion (SNF) [4] fuses multiple networks to one network by iteratively updating a sequence of nonnegative status matrices. The third class of methods aim to learn an optimal linear combination of multiple kernels or similarities [15–20]. For example, the optimized kernel k-means [16] is proposed to obtain optimal linear combination of multiple kernels and cluster assignment matrix simultaneously by minimizing a trace clustering loss.

However, almost all the existing methods assume strong consistency among different views or omics, and thus they capture the clustering structure by using the hidden shared information. This may face problem in the case when the different views share relatively weak common clustering structure. For instance, different views may have different levels of noisy information. Furthermore, different views may have conflicting clustering structures, or one single view may have different clustering structures with all the others. All of these may make it difficult to identify the shared information among views. A biological example is that, the analysis on different omics for glioblastoma multiforme (GBM), an aggressive adult brain tumor, obtains different results. One work [21] based on expression and copy-number-variant data, identifies two subtypes, which is inconsistent with the results obtained in [22], which identifies four subtypes primarily only by expression data. Therefore, when the consistent information is weaker than the conflicting information, which is highly likely in subtype identification, it is challenging to discover the hidden clustering structures. A natural idea to overcome this challenge is to decompose the information in each view to a shared part across all views and a view-specific part. A kernel based method [23] is developed following this idea, which attempts to construct a consensus kernel using multi-omics data. However, for applications, it focuses more on the common part, but ignores the view-specific clustering structure. Furthermore, the semi-definite programming for the optimization problem is computational complex.

In this work, we propose a novel integrative subspace clustering method by assuming that the common structure information is weak across views. The main idea is to find a specific subspace for each view, so that the new representation for each sample in each view in this subspace is a concatenation of two vectors, say, a common representation among all views, and a specific representation for this view. This could make sure that the common parts and the specific parts lie in two orthogonal subspaces for each view. Furthermore, the representations of the common part are expected to be independent with those of each specific part, where the dependence is measured by Hilbert Schmidt Independence Criterion (HSIC). Our main contributions in this work are summarized as follows.

- We propose a novel subspace learning model to discover the common and specific representations for each sample, especially for the case when the common information might be relatively weaker than the specific information. We propose an algorithm to solve the corresponding optimization problem efficiently.
- We test our method on simulation datasets, text multi-view datasets, cancer type identification, and it works the best for most cases. Especially, our model works even the common information across views is very weak.
- We apply the proposed clustering method on subtype identification, by assuming that the subtype information may also come from the view-specific part of a single omics data. We apply our approach to identify subtypes for five cancers using TCGA datasets. The survival analysis on the clustering results shows that our method works the best for most cases.

## Methods

In this section, we will present the proposed integrative subspace clustering method by multi-view matrix decomposition. We first give a problem statement, and then propose a subspace learning method by mult-view matrix decomposition. We then introduce the Hilbert Schmidt Independence Criterion, and finally propose our integrative subspace clustering model ISC and the corresponding optimization algorithm.

### Problem statement

Suppose we are given *n* samples with *V* views, *X*=[*X*_1_,⋯,*X*_*V*_], where $\phantom {\dot {i}\!}X_{v} \in R^{p_{v} \times n},v=1,\cdots,V$. Denote $X_{v} = [x^{v}_{1},\cdots,x^{v}_{n}]$, where $x^{v}_{i}\in R^{p_{v}}$. The aim is to cluster the *n* samples with a given cluster number based on the integrative information from the *v* views. In cancer subtype identification, the views can be different data sources, omics or platforms.

### Subspace learning for common and specific decomposition

We consider the samples $\phantom {\dot {i}\!}X_{v}\in R^{p_{v}\times n}$ from view *v* are approximately lying in a *d*-dimensional subspace $\Omega _{v}\subset R^{p_{v}}$ (*d*<*p*_*v*_), which is spanned by the columns of an orthonormal matrix $P_{v}\in R^{p_{v}\times d}, P_{v}^{T}P_{v} = I_{d}$. This means that

$$x_{i}^{v} \approx P_{v}z_{i}^{v}, $$

where $z_{i}^{v}\in R^{d}$ is the new representation of $x_{i}^{v}$ in this subspace. We assume that the samples *X*_*v*_ from view *v* have both common and specific clustering structures, which means that $z_{i}^{v}$ can be further represented as

$$z_{i}^{v} = \left(\begin{array}{c} c_{i} \\ s_{i}^{v} \end{array} \right) $$

where $\phantom {\dot {i}\!}c_{i}\in R^{d_{0}}$ is the common representation of *x*_*i*_ across all views, and $s_{i}^{v}\in R^{d_{v}}$ is the specific representation of *x*_*i*_ in the *v*-th view. Note that *d*=*d*_0_+*d*_*v*_. In other words, $x_{i}^{v}$ can be approximately represented as

$$x_{i}^{v} \approx P_{v}z_{i}^{v} \,=\, P_{v}\left(\begin{array}{c} c_{i} \\ s_{i}^{v} \end{array} \right) = \left(P_{v}^{(c)} \ \ P_{v}^{(s)}\right)\left(\begin{array}{c} c_{i} \\ s_{i}^{v} \end{array} \right) =P_{v}^{(c)}c_{i} +P_{v}^{(s)}s_{i}^{v}, $$

where $P_{v} = \left (P_{v}^{(c)} \ \ P_{v}^{(s)}\right), (P_{v}^{(c)})^{T}P_{v}^{(c)}=I_{d_{0}}$ and $\left (P_{v}^{(s)}\right)^{T}P_{v}^{(s)}=I_{d_{v}}$. This means that the *d*-dimensional subspace *Ω*_*v*_ spanned by *P*_*v*_ is further decomposed to two orthogonal subspaces $\Omega _{v}^{(c)}$ and $\Omega _{v}^{(s)}$, spanned by orthonormal matrices $P_{v}^{(c)}$ and $P_{v}^{(s)}$, respectively. In other words, $\Omega _{v} = \Omega _{v}^{(c)} \oplus \Omega _{v}^{(s)}$, where $\Omega _{v}^{(c)}$ and $\Omega _{v}^{(s)}$ are orthogonal subspaces to each other. We can rewrite the above equations in a matrix form as follows,

1 $$ \begin{array}{lll} X_{v}& = & P_{v} Z_{v} +E_{v} \\ &= & \left(P_{v}^{(c)} \ \ P_{v}^{(s)}\right) \left(\begin{aligned} C \\ S_{v} \end{aligned} \right) + E_{v}\\ & = & P_{v}^{(c)}C+P_{v}^{(s)}S_{v} +E_{v}\\ &= & P_{v} \left(\begin{aligned} C \\ S_{v} \end{aligned} \right) + E_{v}, \ \ v=1,\cdots,V \\ \end{array}  $$

where $Z_{v}=\left [z_{1}^{v},\cdots,z_{n}^{v}\right ], C=\left [c_{1},\cdots,c_{n}\right ], S_{v}=\left [s_{1}^{v},\cdots,s_{n}^{v}\right ]$, and *E*_*v*_ is the error matrix for view *v*.

**Fig. 1:**
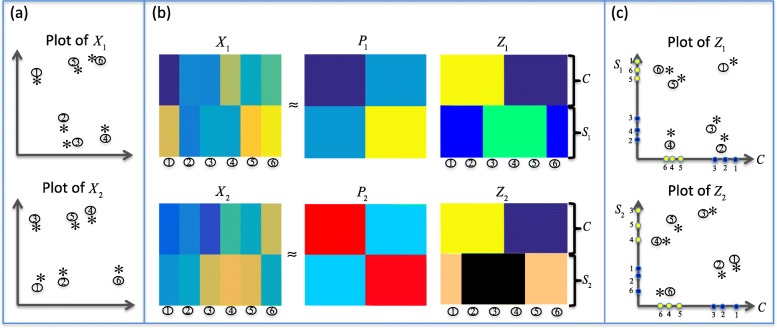
Demonstration of the main idea for the common and specific decomposition in our ISC model. **a** shows the plots for *X*_1_ and *X*_2_ respectively. **b** shows how the original *X*_*v*_ is decomposed to two parts *C* and *S*_*v*_ in two subspaces. **c** shows the plots for the reconstructed *Z*_*v*_, respectively. Note that the two axes of *Z*_*v*_ represent two subspaces. We can see that in the two subspaces, the samples are clustered in different ways

We demonstrate the decomposition idea in Fig. 1. We attempt to find two orthogonal subspaces $\Omega _{v}^{(c)}$ and $\Omega _{v}^{(s)}$ for each view *v*, such that *X*_*v*_ could be decomposed to the common part *C* and the specific part *S*_*v*_ in the subspace $\Omega _{v} = \Omega _{v}^{(c)} \oplus \Omega _{v}^{(s)}$. Hopefully, the common clustering structure is hidden in *C*, and the specific clustering structure for view *v* is hidden in *S*_*v*_.

### Hilbert-Schmidt Independence criterion (HSIC)

To better decompose each view to a common and a view-specific part, such that each view-specific clustering structure in *S*_*v*_ is independent to the common part *C* across all views, a measurement for independence is required. We measure the independence by using the Hilbert-Schmidt Independence Criterion (HSIC) which is a measure of statistical independence [24]. Intuitively, HSIC can be considered as a squared correlation coefficient between two random variables *c* and *s* computed in feature spaces $\mathcal {F}$ and $\mathcal {G}$.

Let *c* and *s* be two random variables from the domains $\mathcal {C}$ and $\mathcal {S}$, respectively. Let $\mathcal {F}$ and $\mathcal {G}$ be feature spaces on $\mathcal {C}$ and $\mathcal {S}$ with associated kernels $ k_{c}: \mathcal {C} \times \mathcal {C} \rightarrow \mathbb {R}$ and $k_{s}: \mathcal {S} \times \mathcal {S} \rightarrow \mathbb {R}$, respectively. Denote the joint probability distribution of *c* and *s* by *p*_(*c*,*s*)_, and (*c*,*s*) and (*c*^′^,*s*^′^) are drawn according to *p*_(*c*,*s*)_. Then the Hilbert Schmidt Independence Criterion can be computed in terms of kernel functions via:

$$\begin{array}{*{20}l} \text{HSIC}(p_{(c,s)},\mathcal{F},\mathcal{G})&=\mathbf{E}_{c,c',s,s'}[k_{c}(c,c')k_{s}(s,s')]\notag\\ &\quad+\mathbf{E}_{c,c'}[k_{c}(c,c')]\mathbf{E}_{s,s'}[k_{s}(s,s')] \notag\\ &\quad-2\mathbf{E}_{c,s}[\mathbf{E}_{c'}[k_{c}(c,c')]\mathbf{E}_{s'}[k_{s}(s,s')]], \end{array} $$

where **E** is the expectation operator.

The empirical estimator of HSIC for a finite sample of points *C* and *S* from *c* and *s* with *p*_(*c*,*s*)_ was given in [24] to be

2 $$\begin{array}{*{20}l} \text{HSIC}((C,S),\mathcal{F},\mathcal{G})\propto tr({K_{c}HK_{s}H}), \end{array} $$

where *tr* is the trace operator of a matrix, *H* is the centering matrix $H = I_{n}-\frac {ee^{T}}{n}$ (e is a proper dimensional column vector with all ones), and *K*_*c*_ and *K*_*s*_∈*R*^*n*×*n*^ are kernel matrices. The smaller the HSIC value, the more likely *C* and *S* are independent from each other.

### Integrative subspace clustering (ISC) model

Based on the above considerations, we propose our integrative subspace clustering model as follows,

3 $$ {\begin{aligned} \begin{array}{l} \min_{\substack{P_{1},\cdots,P_{V}\\ C,S_{1},\cdots,S_{V}}} \sum_{v=1}^{V} \left|\left| X_{v}-P_{v} \left(\begin{aligned} C \\ S_{v} \end{aligned} \right) \right|\right|_{F}^{2} +\beta \sum_{v=1}^{V} tr\left(C^{T}CHS_{v}^{T}S_{v}H\right) \\ s.t.~ P_{v}^{T}P_{v}=I, \end{array}  \end{aligned}}  $$

where $S_{v}^{T}S_{v}$ and *C*^*T*^*C* are the linear kernels of *S*_*v*_ and *C*, respectively, and *β* is a parameter. Note that the first term is the decomposition term that tries to find the orthogonal subspaces where the corresponding common and view-specific representations lie in, and the second independence term is to minimize the dependence between the common part and the view-specific part. We use the linear kernel of *C* and *S*_*v*_ to simplify the computation. After *C* and *S*_*v*_s for all views are obtained, *k*-means clustering is applied to cluster the samples represented by *C* and *S*_*v*_, respectively. The clustering results by using the common part *C* and the specific part *S*_*v*_ are called ISC-C, ISC-S1,ISC-S2, ⋯, respectively.

Based on the resulting *C* and *S*_*i*_s, we define a consensus score(C-score) which is similar to [23] as below:

4 $$ \text{C-score}_{i}=\frac{tr\left(HX_{i}^{T}X_{i}HC^{T}C\right)}{tr\left(HX_{i}^{T}X_{i}H\left(C^{T}C+S_{i}^{T}S_{i}\right)\right)}.   $$

C-score is used to measure the weight of the consensus part in the *i*-th view. Note that the C-score ranges from 0 to 1, and a higher C-score implies stronger consistent information in the corresponding view.

### Optimization algorithm

We propose an alternative updating approach to solve the optimization problem (3).

Step 1. We first fix *P*_*v*_ and *C* in (3), and solve for optimal *S*_1_,⋯,*S*_*v*_ one by one. The *v*-th optimization subproblem can be written as:

5 $$ \min_{S_{v}} \left|\left| X_{v}-P_{v} \left(\begin{aligned} C \\ S_{v} \end{aligned} \right) \right|\right|_{F}^{2}+\beta tr(C^{T}CHS_{v}^{T}S_{v}H).   $$

Since *P*_*v*_ can be represented as $P_{v}=(P_{v}^{(c)} \ \ P_{v}^{(s)})$, the subproblem (5) to solve for *S*_*v*_ can be simplified to:

6 $$ {\begin{aligned} \begin{array}{ll} \min\limits_{S_{v}}& tr\left(-2 X_{v}^{T} P_{v}^{(s)} S_{v} + 2 S_{v}^{T} \left(P_{v}^{(s)}\right)^{T} P_{v}^{(c)} C + S_{v}^{T} \left(P_{v}^{(s)}\right)^{T} P_{v}^{(s)} S_{v}\right) \\ & +\beta tr\left(C^{T}CHS_{v}^{T} S_{v}H\right) \end{array}  \end{aligned}}  $$

By setting the derivatives of the objective function *f*(*S*_*v*_) in (6) with respect to *S*_*v*_ to be zero, we obtain

7 $$ {\begin{aligned} \frac{\partial f(S_{v})}{\partial S_{v}}&=0 \Rightarrow \left(P_{v}^{(s)}\right)^{T} P_{v}^{(s)}S_{v}+\beta S_{v}H C^{T}C H \\ &= \left(P_{v}^{(s)}\right)^{T}X_{v}-\left(P_{v}^{(s)}\right)^{T} P_{v}^{(c)}C. \end{aligned}}  $$

The matrix equation for *S*_*v*_ in (7) is a standard Sylvester equation and can be solved efficiently using method in [25].

Step 2. We then fix *C*,*S*_1_,⋯,*S*_*V*_, and solve the optimization problem (3) for optimal *P*_1_,⋯,*P*_*V*_ one by one. The corresponding *v*-th optimization subproblem can be written as:

8 $$ \min_{P_{v}} \left|\left| X_{v}-P_{v} Z_{v} \right|\right|_{F}^{2} \qquad \quad s.t.\quad P_{v}^{T}P_{v}=I,   $$

where $Z_{v} = \left (\begin {aligned} C \\ S_{v} \end {aligned} \right).$ The optimization problem (8) is a least square problem on grassman manifold, and solved by algorithm 2 in [26].

Step 3. We fix *P*_1_,⋯,*P*_*V*_ and *S*_1_,⋯,*S*_*V*_, then solve the optimization problem (3) for *C*. The corresponding subproblem can be written as:

9 $$ {\begin{aligned} \begin{array}{ll} \min\limits_{C} & \sum_{v}^{V} tr\left(-2 X_{v}^{T} P_{v}^{(c)}C +2 S_{v}^{T} \left(P_{v}^{(s)}\right)^{T} P_{v}^{(c)} C + C^{T} \left(P_{v}^{(c)}\right)^{T} P_{v}^{(c)} C\right) \\ & + \beta tr\left(S_{v}^{T} S_{v} H C^{T} C H \right). \end{array}  \end{aligned}}  $$

Similarly, we set the derivatives of objective function of the subproblem (9) with respect to *C*, and obtain

10 $$ {\begin{aligned} \left(\sum_{v=1}^{V} \left(P_{v}^{(c)}\right)^{T} P_{v}^{(c)}\right) C &+ \beta C \left(\sum_{v=1}^{V} HS_{v}^{T}S_{v}H\right)\\ &=\sum_{v=1}^{V}{ \left(P_{v}^{(c)}\right)^{T} X_{v}-\left(P_{v}^{(c)}\right)^{T} P_{v}^{(s)} S_{v} }.  \end{aligned}}  $$

The matrix equation for *C* in (10) is also a standard Sylvester equation and the same algorithm for solving (7) can be used.

**Fig. 2:**
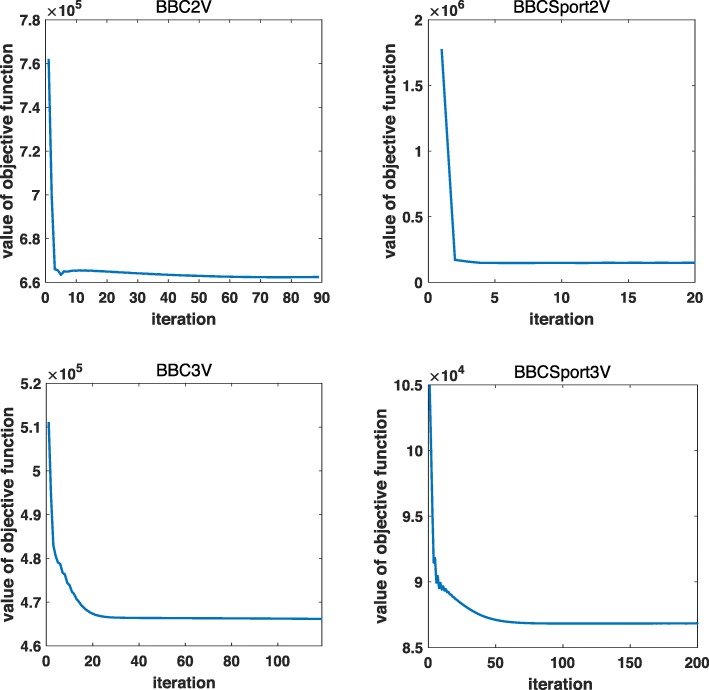
Convergence of the objective values of our algorithm on four datasets of BBC2V, BBC3V, BBCSport2V and BBCSport3V


The overall algorithm for solving (3) is shown in the algorithm box ISC. For each iteration, we need to solve three subproblems in our ISC algorithm to alternatively update *S*_*v*_,*P*_*v*_ and *C*. Since the objective function of ISC model in (3) has a lower bound of zero. and the objective values of our method is decreasing at each step to solve the three subproblems. Therefore the convergence of objective values in our algorithm can be assured. We also experimentally show the convergence of objective values by using four text datasets in Fig. 2, which further confirms the convergence analysis above.

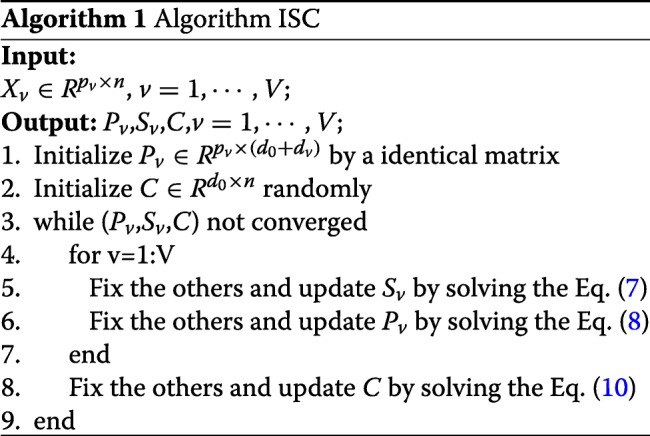


## Results

### Comparative methods

We compare our ISC model with the following comparative methods.

- Spectral clustering for single views(SV1, SV2).
- Co-regularized spectral clustering (Coreg) [3]. The coreg method extends the single view spectral clustering method by adding a co-regularization term which forces the low embeddings from multiple views to be close.
- Similarity network fusion (SNF) [4]. The SNF method integrates the sample similarity network constructed by each data type into a single similarity network by a nonlinear combination approach. This converged network can be used to cluster multi-view datasets.
- Enhanced consensus multi-view clustering model(ECMC) [23]. The ECMC method attempts to find the consensus kernels of multiple views by dividing the kernel of each view into a consensus kernel and a disagreement kernel. The method can achieve a relatively good clustering effects even the correlation between views is weak.

### Measurements of clustering performance

We use the following three measurements to evaluate the clustering results when the ground truth clustering is given.

- Normalized mutual information (NMI). The normalized mutual information (NMI) of a clustering result ${\mathcal {C}} = \{C_{k}\}$ is defined as
  $$\begin{array}{*{20}l}  \mbox {NMI}({\mathcal{C}},{\mathcal{C}}^{*})=\frac{2\text{MI}({\mathcal{C}},{\mathcal{C}}^{*})}{H({\mathcal{C}})+H({\mathcal{C}}^{*}) }\quad \text{with}\quad \\  \text{MI}({\mathcal{C}},{\mathcal{C}}^{*}) = \sum_{C_{k}\in {\mathcal{C}},C_{\ell}^{*} \in {\mathcal{C}}^{*}} p\left(C_{k},C_{\ell}^{*}\right)\cdot \log_{2} \frac{p\left(C_{k},C_{\ell}^{*}\right)}{p(C_{k})p\left(C_{\ell}^{*}\right)}, \end{array} $$
  where ${\mathcal {C}}^{*}=\{C^{*}_{l}\}$ is the ground truth clustering, $p(C_{k}):= |C_{k}|/n, p\left (C_{i},C_{j}^{*} \right)$ is the joint probability of the two classes *C*_*i*_ and $C_{j}^{*}$, and $H({\mathcal {C}}) = -\sum _{C_{i} \in {\mathcal {C}}}p(C_{i})\log _{2} (p(C_{i})).$
- Average clustering accuracy (ACC). with the clustering labels {*l*_*j*_} of ${\mathcal {C}}$ in a suitable clustering ordering which matches the ground truth labels $\left \{l^{*}_{j}\right \}$ of ${\mathcal {C}}^{*}$, the average clustering correction (ACC) is defined as
  $$ ACC({\mathcal{C}},{\mathcal{C}}^{*}) = \frac1n \sum_{j=1}^{n} \delta\left(l_{j},l_{j}^{*}\right), $$
  where the function $\delta (l_{j},l_{j}^{*})=1$ if $l_{j}=l_{j}^{*}$, or $\delta \left (l_{j},l_{j}^{*}\right)=0$ otherwise.
- Adjusted rand index (ARI). For a computed cluster *C*_*i*_ and a ground truth cluster $C^{*}_{j}$, let $n_{i.}=|C_{i}|, n_{.j}=|C^{*}_{j}|$, and $n_{ij}=|C_{i} \cap C^{*}_{j}|$. The adjusted rand index is defined as
  $$ARI=\frac{RI-E(RI)}{max(RI)-E(RI)},$$
  where $RI=\sum _{i,j} C_{n_{ij}}^{2}, max(RI)=\frac {1}{2}\left (\sum _{i} C_{n_{i.}}^{2}+ \sum _{j} C_{n_{.j}}^{2}\right)$, and $E(RI)=\left (\sum _{i} C_{n_{i.}}^{2}\right)\left (\sum _{j}C_{n_{.j}}^{2}\right)/C_{n}^{2}$, where *C* represents combination number operator. The range of ARI is from -1 to 1. A larger value of ARI means that the clustering result is more consistent with the ground truth clustering.
- Silhouette score (S-score) [27]. When the ground truth clustering is unkonwn, the above criterions could not be computed, and thus Silhouette score defined as follows can be used
  $$\text{S-score} = \frac{1}{n} \sum_{i} \frac{b_{i}-a_{i}}{max\{a_{i},b_{i}\}},$$
  where *a*_*i*_ is the average Euclidean distance from sample *i* to the other samples within the same cluster of sample *i* and *b*_*i*_ is the minimum of the average Euclidean distance from sample *i* to all samples in any one of the other clusters different from the cluster of sample *i*. The range of silhouette score is from -1 to 1. The larger the silhouette score is, the better the clustering structure is.

### Simulation experiments

In this section, we use synthetic datasets to evaluate our ISC model. The synthetic datasets are generated in the following way. We first sample 200 two-dimensional points evenly from a mixed Gaussian distribution with *μ*_1_=[−4,6], *μ*_2_=[3,−10] and a common covariance matrix *Σ*=[10 0;0 6], and thus could obtain a matrix *Y*∈*R*^2×200^. By adding white noises to *Y*, we can get two data matrices *Y*_1_∈*R*^2×200^ and *Y*_2_∈*R*^2×200^, which can be considered as the common part for two views. We then construct two specific matrices *T*_1_ and *T*_2_ by randomly permuting the columns of *Y*_1_ and *Y*_2_, respectively. Finally, we randomly construct two matrices *P*_*v*_∈*R*^8×4^ and construct the two-view matrices *X*_*v*_=*P*_*v*_[*Y*_*v*_;*tT*_*v*_]∈*R*^8×200^,(*v*=1,2), where *t* is a parameter which could control the degree of inconsistency of different views. Note that the ground truth clustering labels for both common part, and the two specific parts are both known and denoted by *y*,*y*_1_,*y*_2_. We construct 10 corresponding datasets by taking *t*={0.1,0.9,1,2,5,6,10,15,20,30}. We report the consensus scores for two views on simulation datasets in Table 1. From the table, we can see that simulation datasets with small *t* have high consensus scores and those with large *t* have low consensus scores.

**Table 1:** Consensus scores and Silhouette scores for the simulation datasets

	Views/Methods	*t*=0.1	*t*=0.9	*t*=1	*t*=2	*t*=5	*t*=6	*t*=10	*t*=15	*t*=20	*t*=30
C-score	V1	0.9998	0.3974	0.2971	0.0244	1.45e-03	9.66e-04	3.50e-04	1.63e-04	9.48e-05	4.38e-05
	V2	0.9999	0.4034	0.3033	0.0233	5.26e-04	2.61e-04	4.94e-05	1.90e-05	1.08e-05	5.13e-06
S-score	ISC-C	**0.890**	**0.936**	**0.920**	**0.895**	**0.937**	**0.937**	**0.889**	**0.939**	**0.889**	**0.888**
	ISC-S1	0.639	0.660	0.671	0.718	0.753	0.754	0.759	0.761	0.762	0.764
	ISC-S2	0.819	0.749	0.761	0.832	0.853	0.854	0.857	0.858	0.858	0.858

The highest silhouette scores are marked in bold

We first compare the three clustering results obtained by our method and show their performance when *t* changes. We apply our ISC model to compute the corresponding common part *C* and the specific parts *S*_1_ and *S*_2_. *k*-means clustering is then applied on *C*, *S*_1_ and *S*_2_, and three corresponding clustering results ISC-C, ISC-S1 and ISC-S2 are obtained, respectively. Since the *k*-means method may be sensitive to the initials, we run the *k*-means method 100 times and report the average of the results. We choose the parameter *β* from {0,1*e*−6,1*e*−5,⋯,1*e*+5,1*e*+6}. We report the average Silhouette scores for the three clustering results in Table 1. As we can see, the clustering result of ISC-C achieves a higher silhouette score than the clustering results of ISC-S1 and ISC-S2 for any *t*, which indicates that the common part may have better clustering structure in the simulation datasets. We also compute the NMI, ACC and ARI by comparing the three clustering results with the ground truth labels *y*,*y*_1_ and *y*_2_, respectively. The average values are reported in Table 2. We have two observations from the results. First, ISC-C peforms perfect when *t* changes, and the results by ISC-S1 and ISC-S2 are getting better when *t* increases. This means that the our ISC-C could always capture the common structure even the consisitency is very weak, and our ISC-S1 and ISC-S2 could capture the specific structures better when the consistency gets weak. Second, ISC-C achieves higher NMI, ACC and ARI values than ISC-S1 and ISC-S2, which is consistent with the results obtained by silhouette scores. This implies that Silhouette scores may be used to select the best clustering result.

**Table 2:** The average NMIs, ACCs and ARIs obtained by the our ISC method and other comparison partners in simulation datasets

	Methods	*t*=0.1	*t*=0.9	*t*=1	*t*=2	*t*=5	*t*=6	*t*=10	*t*=15	*t*=20	*t*=30
NMI	SV1	0.368	0.012	0.003	0.005	0.019	0.020	0.024	0.023	0.023	0.023
	SV2	**1.000**	0.009	0.006	0.001	0.004	0.005	0.006	0.006	0.006	0.006
	Coreg	0.701	0.072	0.039	0.005	0.007	0.006	0.010	0.012	0.010	0.012
	SNF	**1.000**	**1.000**	**1.000**	0.960	0.592	0.161	0.000	0.000	0.000	0.000
	ECMC	**1.000**	0.203	0.051	0.006	0.016	0.020	0.019	0.024	0.023	0.023
	ISC-C	**1.000**	**1.000**	**1.000**	**1.000**	**1.000**	**1.000**	**1.000**	**1.000**	**1.000**	**1.000**
	ISC-S1	0.004	0.301	0.390	0.673	0.806	0.806	0.759	0.759	0.736	0.736
	ISC-S2	0.005	0.756	0.816	0.862	0.889	0.889	0.889	0.889	0.889	0.889
ACC	SV1	0.840	0.563	0.530	0.540	0.580	0.582	0.590	0.590	0.590	0.590
	SV2	**1.000**	0.555	0.545	0.515	0.535	0.540	0.545	0.545	0.545	0.545
	Coreg	0.945	0.655	0.615	0.540	0.550	0.545	0.558	0.565	0.560	0.565
	SNF	**1.000**	**1.000**	**1.000**	0.995	0.900	0.730	0.505	0.505	0.505	0.505
	ECMC	**1.000**	0.663	0.599	0.535	0.575	0.582	0.579	0.588	0.586	0.587
	ISC-C	**1.000**	**1.000**	**1.000**	**1.000**	**1.000**	**1.000**	**1.000**	**1.000**	**1.000**	**1.000**
	ISC-S1	0.537	0.810	0.850	0.940	0.970	0.970	0.960	0.960	0.955	0.955
	ISC-S2	0.540	0.955	0.970	0.980	0.985	0.985	0.985	0.985	0.985	0.985
ARI	SV1	0.460	0.011	-0.001	0.001	0.021	0.022	0.028	0.028	0.028	0.028
	SV2	**1.000**	0.007	0.003	-0.004	-0.000	0.001	0.003	0.003	0.003	0.003
	Coreg	0.791	0.092	0.048	0.001	0.005	0.003	0.009	0.012	0.009	0.012
	SNF	**1.000**	**1.000**	**1.000**	0.980	0.638	0.208	-0.004	-0.004	-0.004	-0.004
	ECMC	**1.000**	0.229	0.063	0.003	0.018	0.022	0.021	0.028	0.027	0.027
	ISC-C	**1.000**	**1.000**	**1.000**	**1.000**	**1.000**	**1.000**	**1.000**	**1.000**	**1.000**	**1.000**
	ISC-S1	0.001	0.381	0.487	0.773	0.883	0.883	0.846	0.846	0.827	0.827
	ISC-S2	0.001	0.827	0.883	0.921	0.941	0.941	0.941	0.941	0.941	0.941

The highest NMIs, ACCs and ARIs are marked in bold

**Fig. 3:**
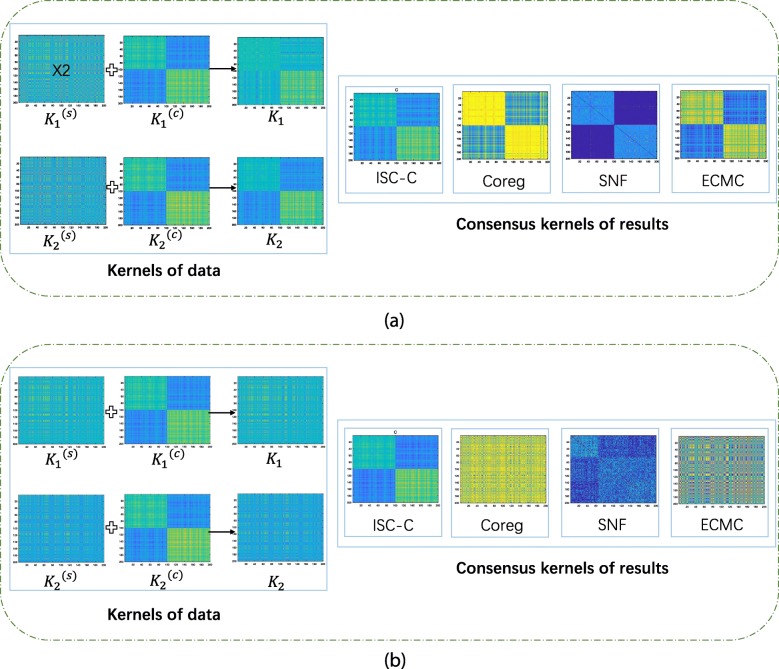
Performance comparison between ISC-C and other methods on simulation datasets with **a***t*=0.1 and **b***t*=10

We then compare our clustering result by ISC-C with the comparison methods by computing NMI, ACC, and ARI of each methods, which all assume strong consistency across views except ECMC. The average values of all the methods are reported in Table 2. When *t* is relatively small, almost all the methods could perform well. When the degree of inconsistency increases as *t* increases, our method ISC-C outperforms other methods. That is because, when the consistency signal is very weak, existing methods could not capture the common clustering structure any more, but our ISC-C could discover the common clustering structure very well. We also plot the clustering results for all multi-view methods with *t*=0.1 and *t*=10 in Fig. 3. In the figure, since the common result of the SNF method is in the form of the kernel, we present all the data in the form of a kernel. Specifically, as for the simulation datasets, the linear kernel of *X*_*v*_,*Y*_*v*_ and *T*_*v*_ are denoted as $K_{v}, K_{v}^{c}$ and $K_{v}^{s}$, respectively. In addition, when using a linear kernel, equations $K_{v}=K_{v}^{c}+K_{v}^{s}$ hold for *v*=1,2. We can see that in Fig. 3a, *t* is small and consensus score is big, and all methods could discover the latent common clustering structure with high accuracy. However, in Fig. 3b, when *t* is big and the consensus score is low, all baseline methods fail to discover the best clustering structure, but our ISC-C method could still capture the common structure across views. This further shows the power of our method even when the common information is very weak.

### Experiments on multi-view text datasets

In this section, we evaluate our ISC method on multi-view text datasets. Since only the ground truth labels for common part is known, we compare the ISC-C results with other methods.

- BBC and BBCSport datasets. BBC datasets consist of 2,225 documents provided by the BBC News website, which are stories about the five thematic areas of business, entertainment, politics, sports and technology from 2004 to 2005. The BBCSport datasets consist of 737 documents from the BBC Sports website, which correspond to sports news articles in the five subject areas of sports, cricket, football, rugby and tennis from 2004 to 2005. Each article is divided into up to four parts, each part has at least 200 characters, and then the pieces are randomly assigned to each view, which can generate the dataset of BBC2/3/4views and BBCSport2/3/4views. Here we only select BBC2/3views, BBCSport2/3views datasets for clustering.
- Cora dataset. The Cora dataset consists of machine learning papers that are one of seven categories: case-based, genetic algorithms, neural networks, probabilistic methods, reinforcement learning, rule learning, and theory. There are 2,708 papers in the entire corpus. The dataset consists of two views. One view is represented by a 0/1 value word vector, indicating the absence/presence of the corresponding word in the dictionary. The other view is the citation relationship between each publication and other publications.

By using the ISC model, we could obtain the common part *C*. We then apply k-means clustering on *C*. We compare the results of ISC-C with other methods, and the results are shown in Table 3. We can see from the table that, our ISC model works the best for most cases.

**Table 3:** The average NMIs, ACCs, ARIs and standard errors obtained by the ISC and other comparison partners on text datasets

	Methods	BBC2V	BBC3V	BBCSport2V	BBCSport3V	Cora
NMI	SV1	0.004 ±0.000	0.007 ±0.000	0.067 ±0.005	0.032 ±0.000	0.124 ±0.001
	SV2	0.006 ±0.000	0.007 ±0.000	0.037 ±0.001	0.064 ±0.000	0.010 ±0.000
	SV3	——	0.007 ±0.000	——	0.093 ±0.001	——
	Coreg	0.007 ±0.004	0.062 ±0.031	0.139 ±0.004	0.146 ±0.027	0.102 ±0.008
	SNF	0.307 ±0.002	0.147 ±0.001	0.303 ±0.001	0.119 ±0.004	0.273 ±0.001
	ECMC	——	——	——	**0.373 ±0.002**	——
	ISC-C	**0.397** ±**0.000**	**0.328** ±**0.006**	**0.486** ±**0.001**	0.324 ± 0.005	**0.305** ±**0.001**
ACC	SV1	0.241 ±0.000	0.260 ±0.000	0.376 ±0.002	0.345 ±0.000	0.313 ±0.002
	SV2	0.242 ±0.000	0.249 ±0.000	0.369 ±0.000	0.386 ±0.000	0.251 ±0.000
	SV3	——	0.260 ±0.000	——	0.436 ±0.000	——
	Coreg	0.248 ±0.009	0.297 ±0.021	0.443 ±0.002	0.462 ±0.022	0.344 ±0.010
	SNF	0.307 ±0.003	0.357 ±0.002	0.491 ±0.002	0.390 ±0.003	0.430 ±0.005
	ECMC	——	——	——	**0.612 ±0.003**	——
	ISC-C	**0.479** ±**0.004**	**0.443** ±**0.003**	**0.583** ±**0.001**	0.532 ±0.005	**0.508** ±**0.000**
ARI	SV1	0.000 ±0.000	0.021 ±0.002	0.085 ±0.002	0.000 ±0.000	-0.000 ±0.002
	SV2	0.000 ±0.000	0.006 ±0.000	-0.011 ±0.003	0.000 ±0.000	0.039 ±0.000
	SV3	——	——	——	0.000 ±0.000	0.078 ±0.000
	Coreg	0.002 ±0.003	0.125 ±0.013	0.059 ±0.005	0.015 ±0.013	0.060 ±0.030
	SNF	0.105 ±0.001	0.110 ±0.001	0.090 ±0.005	0.027 ±0.002	0.370 ±0.002
	ECMC	——	——	——	0.370 ±0.002	——
	ISC-C	**0.269** ±**0.005**	**0.356** ±**0.004**	**0.194** ±**0.001**	**0.172** ±**0.005**	**0.197** ±**0.005**

The highest NMI, ACCs and ARIs are marked in bold

### Identifying cancer types by colorectal cancer dataset

Tumors may not be diagnosed pathologically, and thus it’s meaningful to determine whether the patient’s specific symptoms are colon cancer or colorectal cancer. We further evaluate our method by identifying colon cancer and colorectal cancer on a colorectal cancer dataset [28]. which consists exome sequences, DNA copy number, promoter methylation and messenger RNA, and microRNA expression for 276 patients. We select three types of expression data including DNA methylation, mRNA expression and miRNA expression. Specifically, DNA methylation profiles are obtained by the Illumina Infinium HumanMethylation27 arrays, mRNA expression profiles are generated by Agilent microarray, and miRNA quantification via Illumina sequencing. After screening, we obtain 85 cancer patients with colon cancer and colorectal cancer.

We apply our ISC model to identify the cancer types (colon cancer or colorectal caner) for these patients with two or three views, and obtain the corresponding common part *C* and three specific parts *S*1,*S*2 and *S*3. Since we assume that the cancer type or subtype structures may be specifically shown in a single omics, we check the clustering results for both the common and specific parts and see whether they capture the clustering information for cancer types. Note that the ground truth for cancer types is known, thus we could also calculate NMI, ACC and ARI by using the common part ISC-C, the specific parts ISC-S1, ISC-S2, ISC-S3. The results are reported in Table 4. Our method performs better than the baseline methods for most of the cases. Overall, our method ISC-C with common part with DNA methylation and miRNA expression data performs the best among all the obtained clustering results. While for miRNA and mRNA expression, SNF works the best, our ISC method with the specific part of DNA methylation (ISC-S1) works the best among all methods on the view combinations with DNA methylation. It may imply that DNA methylation plays an important role in the identification of the cancer type. This confirms our hypothesis that information about the type of cancer may be hidden in a particular omics.

**Table 4:** The average NMIs, ACCs and ARIs and standard errors obtained by the ISC and other comparison partners on colorectal cancer datasets

	Methods	DNA methylation+	DNA methylation+	miRNA expression+	DNA methylation+miRNA
		miRNA expression	mRNA expression	mRNA expression	expression+mRNA expression
NMI	SNF	0.247 ±0.001	0.247 ±0.004	**0.330** ±**0.003**	0.276 ±0.000
	Coreg	0.023 ±0.000	0.186 ±0.000	0.186 ±0.000	0.234 ±0.008
	ECMC	0.164 ±0.000	0.164 ±0.000	0.091 ±0.004	0.138 ±0.006
	ISC-C	**0.372** ±**0.006**	0.149 ±0.001	0.137 ±0.015	0.012 ±0.004
	ISC-S1	0.118 ±0.005	**0.338** ±**0.004**	——	**0.288** ±**0.002**
	ISC-S2	0.019 ±0.002	——	0.046 ±0.007	0.009 ±0.005
	ISC-S3	——	0.175 ±0.002	0.263 ±0.003	0.178 ±0.001
ACC	SNF	0.835 ±0.004	0.800 ±0.006	**0.847** ±**0.003**	0.835 ±0.005
	Coreg	0.812 ±0.000	0.812 ±0.000	0.812 ±0.000	0.812 ±0.000
	ECMC	0.741 ±0.000	0.741 ±0.000	0.642 ±0.005	0.706 ±0.004
	ISC-C	**0.871** ±**0.003**	0.602 ±0.002	0.689 ±0.000	0.567 ±0.004
	ISC-S1	0.698 ±0.004	**0.859** ±**0.004**	——	**0.843** ±**0.006**
	ISC-S2	0.583 ±0.009	——	0.685 ±0.008	0.566 ±0.002
	ISC-S3	——	0.779 ±0.007	0.828 ±0.001	0.757 ±0.003
ARI	SNF	0.391 ±0.003	0.310 ±0.005	**0.442** ±**0.004**	0.402 ±0.004
	Coreg	0.031 ±0.003	0.250 ±0.000	0.250 ±0.000	0.336 ±0.013
	ECMC	0.209 ±0.000	0.209 ±0.000	0.080 ±0.005	0.160 ±0.006
	ISC-C	**0.506** ±**0.001**	-0.007 ±0.009	0.113 ±0.003	0.000 ±0.017
	ISC-S1	0.139 ±0.000	**0.469** ±**0.006**	——	**0.422** ±**0.005**
	ISC-S2	0.015 ±0.002	——	0.098 ±0.008	0.011 ±0.009
	ISC-S3	——	0.238 ±0.005	0.384 ±0.006	0.237 ±0.006

The highest NMIs, ACCs and ARIs are marked in bold

## Applications on cancer subtype identification using TCGA datasets

We finally apply our ISC model on The Cancer Genome Atlas (TCGA) Research Network[29] to identify subtypes for five cancers. TCGA is currently the largest database of cancer genetic information, and has included 33 types of cancer including 10 rare cancer types. In addition, in the database, each cancer data contains gene expression data, miRNA expression data, copy number variation, DNA methylation, SNP, etc., and has sufficient clinical data.

### Data sets

The datasets for five cancers using TCGA datasets are collected by Wang et al. [4]. The datasets contain five cancer types: polymorphism Glioblastoma (GBM), renal clear cell carcinoma (KRCCC), breast invasive carcinoma (BIC), colon adenocarcinoma (COAD) and lung squamous cell carcinoma (LSCC). There are three types of cancer expression data: DNA methylation, mRNA expression, and miRNA expression, as well as clinical information, including survival data for patients. Since we don’t have the ground truth labels for the subtypes of these datasets, survival analysis is mainly used to evaluate our model.

For each of the five datasets, we apply the ISC model to compute the common part and specific parts, and then apply k-means to obtain clustering results. The procedure for obtaining the cancer subtype of the dataset is the same as that of Colorectal cancer dataset. The numbers of subtypes are chosen as 3, 3, 4, 3 and 4 for GBM, KRCCC, BIC, COAD, and LACC[4], respectively. We also report consensus scores for the three views of the five cancers in Table 5. As we can see, the consensus scores for the first two views are both very low. This implies that the consistency information across views are relatively weaker compared to the inconsistency, and thus the traditional multi-view methods may not work.

**Table 5:** Consensus scores of three views for the five TCGA cancer datasets

Cancer types	mRNA expression	miRNA expression	DNA expression
GBM	0.007	0.089	0.102
BIC	0.083	0.028	0.529
KRCCC	0.015	0.022	0.474
LSCC	0.033	0.002	0.402
COAD	0.041	0.005	0.511

### Survival analysis

We apply the log-rank test to measure whether different subtypes obtained by clustering are meaningful, since the survival time in months are given for each sample in the TCGA datasets. The log-rank test is a commonly used non-parametric test method for comparison of survival processes in survival analysis and can be used to compare whether two or more sets of survival curves are identical. In general, the smaller the *p*-value obtained from it, the more different the survival curves of the two or more groups.

The log-rank *p*-values for all the methods are reported in Table 6. we can see from the table that, for four cancers including GBM, BIC, KRCCC, and LSCC, our ISC method could obtain the most significant *p*-values. For COAD, our method with ISC-S2 could obtain the similarly good *p*-value with the ECMC method. Furthermore, the subtypes for GBM and KRCCC found by the common part across three views obtain the most significant *p*-values, the BIC subtypes found by miRNA expression are the most significant, and the subtypes for LSCC found by DNA methylation are the most significant. We also report the silhouette scores for the clustering results of ISC-C, ISC-S1, ISC-S2, and ISC-S3 in Table 7. By comparing Tables 6 and 7, for four of five datasets except GBM, the best clustering results with the best cox *p*-values among our four clustering results are corresponding to the highest silhouette scores. This implies that the our selection sheme for the clustering results is effective in this application.

**Table 6:** Cox *p*-values of survival analysis obtained by different clustering methods for the five cancers in TCGA datasets

Methods	GBM	BIC	KRCCC	LSCC	COAD
mRNA expression	5.67e-01	9.30e-02	9.54e-01	6.00e-03	1.93e-01
DNA Methylation	1.55e-01	5.77e-04	8.11e-01	1.30e-02	1.10e-02
miRNA expression	1.88e-01	9.80e-01	8.34e-01	1.17e-01	7.14e-01
Coreg	2.00e-03	4.81e-05	1.63e-04	5.00e-03	7.00e-03
SNF	8.00e-03	3.46e-05	8.00e-03	1.66e-04	2.00e-03
ECMC	1.70e-02	7.26e-06	1.00e-02	6.95e-04	**3.87e-04**
ISC-C	**3.66e-08**	2.62e-04	**1.04e-04**	9.19e-12	2.11e-02
ISC-S1	4.00e-03	1.44e-03	2.56e-04	8.07e-06	7.68e-03
ISC-S2	8.05e-05	6.12e-05	2.55e-04	**2.67e-13**	7.12e-04
ISC-S3	3.00e-03	**3.28e-06**	1.92e-04	2.45e-04	3.20e-02

The lowest p-values are marked in bold

**Table 7:** Silhouette scores by different clustering methods for the five cancers in TCGA datasets

Methods	GBM	BIC	KRCCC	LSCC	COAD
ISC-C	0.524	0.508	**0.717**	0.570	0.454
ISC-S1	**0.679**	0.585	0.598	0.540	0.570
ISC-S2	0.536	0.580	0.711	**0.783**	**0.579**
ISC-S3	0.530	**0.651**	0.660	0.675	0.556

The highest silhouette scores are marked in bold

**Fig. 4:**
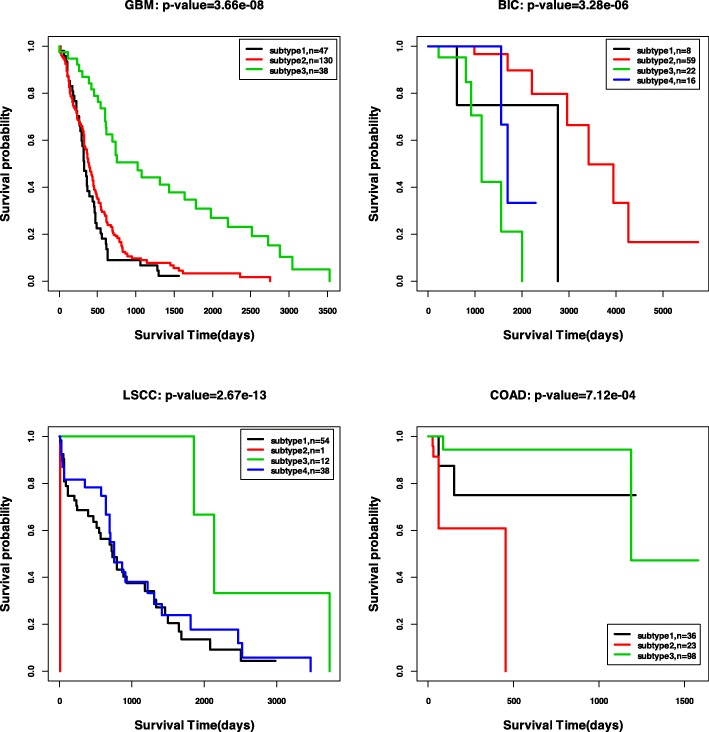
Kaplan-Meier survival curves for the four cancer types (*p*-values are reported in Table 6)

**Fig. 5:**
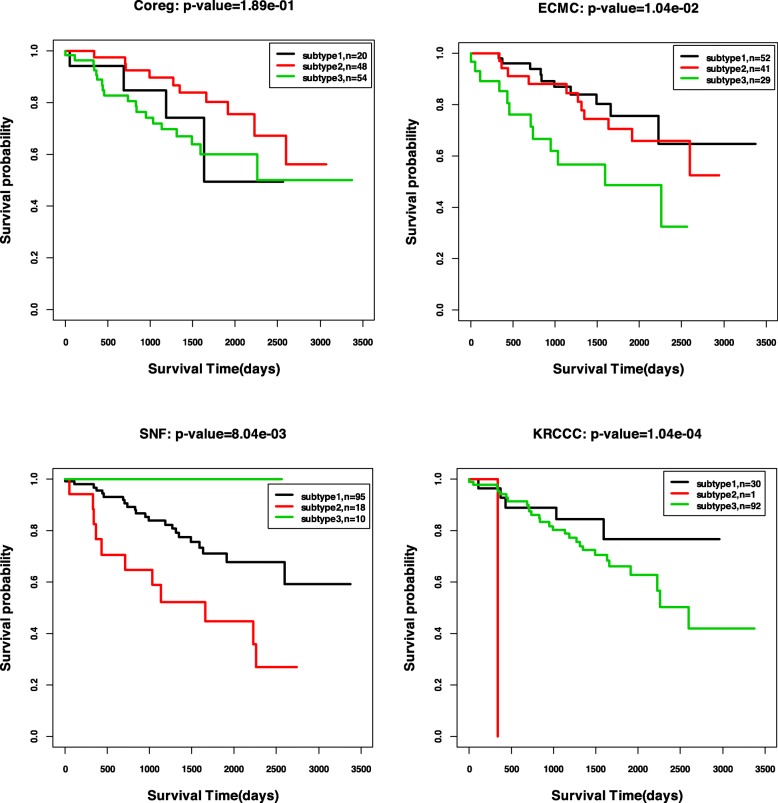
Kaplan-Meier survival curves for KRCCC by four methods: Coreg, ECMC, SNF and ISC (*p*-values are reported in Table 6)

We also plot the Kaplan-Meier survival curves by the ISC clustering results with the most significant *p*-values for all the five cancer types. Figure 4 shows the curves for GBM, BIC, COAD, and LSCC, and Fig. 5 shows the curve for KRCCC. From the figures, we could see the significantly different survival profiles over the subtypes. For the cancer KRCCC, we also plot the Kaplan-Meier survival curves obtained by baseline methods Coreg, ECMC and SNF in Fig. 5. We can see the survival curves by our ISC method are more significantly different than that obtained by the other compared methods.

### Subtype visualization

**Fig. 6:**
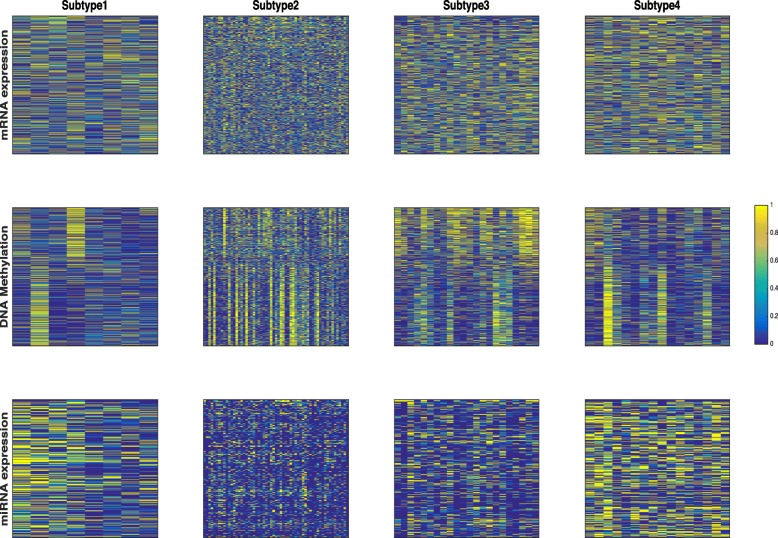
Visualization of the three data types in four subtypes for Breast cancer

We further analyze the obtained breast cancer subtypes by our model ISC with *S*3, since *S*3 by miRNA expression generates the most significantly different survival profiles across different subtypes. Fig. 6 shows the visualization of four breast cancer subtypes identified by the specific part of miRNA (S3). It can be seen that with the clustering results, the samples in the other two views - mRNA expression and DNA methylation- are not separated, and some subtypes are even very similar. However, the characteristics of miRNA expression for the four subtypes seem significantly different. This implies that the resulting best subtype identified by ISC-S3 is specifically shown by miRNA expression, but not shown in other views.

### Drug treatment analysis on cancer subtypes

**Fig. 7:**
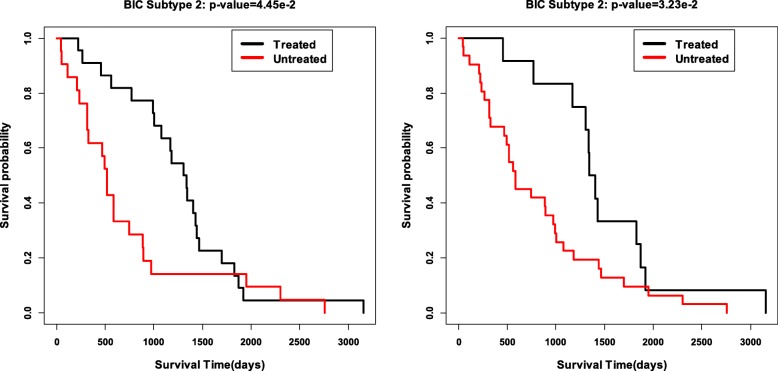
Survival analysis of the treatment with Cytoxan and Adriamycin in Breast cancer Subtype 2 with *p*-values 4.45e-02 and 3.23e-02, respectively

**Fig. 8:**
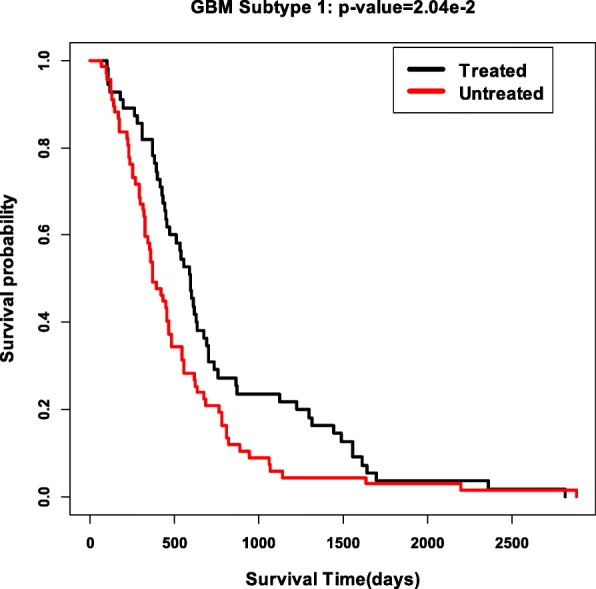
Survival analysis of the Temozolomide treatment in GBM subtype 1 with *p*-value 2e-2

We finally validate the obtained subtypes by comparing the survival profiles from different treatment groups in each subtype. We choose two drug treatments of Cytoxan and Adriamycin for breast cancer, and drug treatment temozolomide for GBM. For each subtype, we check whether the survival profiles are significantly different between the treatment patients and the untreated patients. The Cox *p*-values for all the three treatments in all subtypes are reported in Table 8. Interestingly, we can see that for breast cancer, the patients in Subtype 2 is sensitive to the two drug treatments of Cytoxan and Adriamycin. The Kaplan-Meier survival curves of these two treatments in Subtype 2 are shown in Fig. 7. In Subtype 1 of GBM, the patients with treatment temozolomide have significantly different survival profiles with the untreated patients in this subtype. the Kaplan-Meier survival curves of glio cancers in Subtype 1 is shown in Fig. 8. These further validate that the Subtypes we cound is biological meaningful.

**Table 8:** Survival analysis of three treatments on four BIC subtypes and three GBM subtypes

Treatment	All	Subtype1	Subtype2	Subtype3	Subtype4
Cytoxan(BIC)	3.3e-02	6.1e-01	**4.5e-02**	4.83e-01	5.01e-01
Adriamycin(BIC)	1.3e-02	2.77e-01	**3.2e-02**	8.21e-01	2.66e-01
Temozolomide(GBM)	3.6e-02	**2.0e-02**	9.18e-01	3.88e-01	——

The treatment can significantly improve treatment outcomes in the subtype of p-value in boldface

### Discussion on breast subtypes

We further discuss the subtypes we found for breast cancer. Breast cancer is a heterogeneous and polygenic disease, which is one of the most common malignancies in women. Based on histological and genomic features, breast cancer can be roughly separated into four subtypes (luminal A, luminal B, HER2-amplified, and basal-like) [30].

To date, researchers have reported many genes related to subtypes of breast cancer. We firstly collect genes associated with these subtypes, respectively, and then check the matching between our resulting four subtypes and these four known subytpes. BUB1, CDCA4, CHEK1, FOXM1 and HDAC2 probably are the key genes in basal-like subtype. Because alterations in these genes is a kind of deletion event in the basal cancers, which is related with basal-like cancer enriched subgroup, harbours chromosome 5q deletions, and several signaling molecules, transcription factors and cell division genes [31]. Besides, basal-like subtype may also correlate with the gene EGFR, which is supported with the fact that alterations of EGFR, p53 and pTeN are cooperative and likely to play an important role in basal-like breast cancer pathogenesis[32]. For luminal B subtype, PPP2R2A is an associated gene due to the dysregulation of specific PPP2R2A functions in luminal B breast cancers [31]. The genes ZNF703 and DHRS2 are likely to correlate with luminal B since [33] suggests ZNF703 is a luminal B specific driver and Tumors with elevated ZNF703 levels were characterized by alterations in a lipid metabolism and detoxification pathway that include DHRS2 as a key signaling component. For HER2 subtype, [34] confirms that agents targeting GAB2 or GAB2-dependent pathways may be useful for treating breast tumors that overexpress HER2, and thus we include GAB2 as a correlated gene for HER2 type breast cancer. Besides, Trastuzumab blocks the HER2-HER3(ERBB3) interaction and is used to treat breast cancers with HER2 overexpression, although some of these cancers develop trastuzumab resistance. By using small interfering RNA (siRNA) to identify genes involved in trastuzumab resistance, [35] identified several kinases and phosphatases that were upregulated in trastuzumab-resistant cancers, including PPM1H. This suggests that PPM1H and ERBB3 may have some link with HER2 type breast cancer.

For each computed subtype by our ISC algorithm, we first calculate t-test *p*-values for each of these correlated genes to show whether the gene expression levels are significantly changed between the subtype and the other subtypes. We then apply the Fisher’s combined probability test [36] to compute the group *p*-values for these genes, which could test whether the group of the selected genes are significantly different between the subtype the and other subtypes. We report the group *p*-values for each resulting subtype in Table 9. The results show that, our computed Subtype 2 is highly likely corresponding to the basal-like breast cancer subtype, with group *p*-value being 3.83e-08. Our computed Subtype 4 may also contain the basal-like breast cancer subtype, with group *p*-value being 4.79e-07. Our Subtype 4 probably corresponds to the HER2 breast cancer subtype, with group *p*-value being 4.17e-07, and our Subtype 3 is likely to correspond to the luminal B breast cancer subtype.

**Table 9:** Group *p*-values for three breast cancer subtypes including basal-like, luminal B and HER2

Group *p*-values	Subtype1	Subtype2	Subtype3	Subtype4
Basal-like	1.69e-01	**3.83e-08**	1.50e-02	4.79e-07
Luminal B	2.44e-01	3.91e-02	**1.17e-02**	3.03e-02
HER2	1.09e-01	3.34e-01	5.69e-03	**4.17e-07**

The subtype with p-value in boldface may correspond to a true breast cancer subtype

## Conclusion

Our goal in this work is to discover common and specific information simultaneously from multi-views when the consistency across views is relatively weak, and the specific signal is strong. We propose integrative subspace clustering method (ISC) by common and specific decomposition to find two orthogonal subspaces for each view. To better distinguish the common and view-specific part, we also hope the common part and view-specific part are as independent as possible by using the measurement HSIC. Our simulation experiments, real-world benchmark experiments, cancer type identification by colorectal data, subtype identification for five cancers by TCGA datasets all show that the ISC model outperforms other state-of-art multi-view clustering algorithms. In particular, we find some interesting subtypes in breast cancer and GBM cancer, and the survival analysis shows that the subtypes are biologically meaningful.

## Data Availability

**Multi-view text datasets** were downloaded from http://mlg.ucd.ie/datasets/bbc.html. **Colorectal cancer dataset** was downloaded from http://www.cbioportal.org/study/summary?id=coadread_tcga_pub. **TCGA datasets** were downloaded on 18/4/2017 from http://compbio.cs.toronto.edu/SNF/SNF/Software.html.

## Abbreviations

BIC: Breast cancer
COAD: Colon cancer
GBM: Glio cancer
HSIC: Hilbert Schmidt Independence Criterion
ISC-C: Clustering by the common part in our ISC method
ISC-S: Clustering by the specific part in our ISC method
KRCCC: Kidney cancer
LSCC: Lung cancer
SV: Single view
TCGA: The cancer genome atlas

## References

[1] Tang W, Lu Z, Dhillon I. Clustering with multiple graphs. 2009; 24(4):1016–21. 10.1109/icdm.2009.125.

[2] Chaudhuri K, Kakade S, Livescu K, Sridharan K. Multi-view clustering via canonical correlation analysis. In: International Conference on Machine Learning: 2009. p. 129–36. 10.1145/1553374.1553391.

[3] Kumar A, Rai P, Daumé H. Co-regularized multi-view spectral clustering. In: Advances in Neural Information Processing Systems 24: 25th Annual Conference on Neural Information Processing Systems 2011. Proceedings of a Meeting Held. Granada: 2012. p. 1413–14. http://papers.nips.cc/paper/4360-coregularized-multi-view-spectral-clustering.

[4] Wang B, Mezlini A, Demir F, Fiume M, Tu Z, Brudno M, Haibekains B, Goldenberg A (2014). Similarity network fusion for aggregating data types on a genomic scale. Nat Methods.

[5] Blum A, Mitchell T. Combining labeled and unlabeled data with co-training. In: Proceedings of the Eleventh Annual Conference on Computational Learning Theory: 1998. p. 92–100. 10.1145/279943.279962.

[6] Muslea I, Minton S, Knoblock C (2006). Active learning with multiple views. J Artif Intell Res.

[7] Wang.W, Zhou.Z. A new analysis of co-training. In: Proceedings of the 27th International Conference on Machine Learning (ICML-10): 2010. p. 1135–1142.

[8] Bickel S, Scheffer T. Multi-view clustering. In: ICDM: 2004. p. 19–26. 10.1109/icdm.2004.10095.

[9] Kumar A, III HD. A co-training approach for multiview spectral clustering. In: Proceedings of the 28thInternational Conference on Machine Learning, ICML. Bellevue: 2011. p. 393–400. https://icml.cc/2011/papers/272icmlpaper.pdf.

[10] Xia R, Pan Y, Du L, Yin J. Robust multi-view spectral clu stering via low-rank and sparse decomposition. In: Proceedings of the Twenty-Eighth AAAI Conference on Artificial Intelligence. Québec: 2014. p. 2149–55.

[11] Tang J, Hu X, Gao H, Liu H. Unsupervised feature selection for multi-view data in social media. In: SDM: 2013. p. 270–8. 10.1137/1.9781611972832.30.

[12] Wang H, Nie F, Huang H. Multi-view clustering via joint nonnegative matrix factorization. In: Proceedings of the 13th SIAM International Conference on Data Mining. Austin: 2013. p. 352–60. http://proceedings.mlr.press/v28/wang13c.html.

[13] Gao J, Han j, Liu j, Wang c. Multi-view clustering via joint nonnegative matrix factorization. In: Proceedings of the 13th SIAM International Conference on Data Mining. Austin: 2013. p. 252–60. 10.1137/1.9781611972832.28.

[14] Qianqian S, Chuanchao Z, Minrui P, Xiangtian Y, Tao Z, Juan L, Luonan C. Pattern fusion analysis by adaptive alignment of multiple heterogeneous omics data. Bioinformatics. 2017. 10.1093/bioinformatics/btx176. 10.1093/bioinformatics/btx176 28520848

[15] Lanckriet G, Cristianini N, Bartlett P, El G, Jordan M (2002). Learning the kernel matrix with semi-definite programming. J Mach Learn Res.

[16] Yu S, Tranchevent L, Liu X, Glanzel W (2011). Optimized data fusion for kernel k-means clustering. Pattern Anal Mach Intell IEEE Trans.

[17] Lange T, Buhmann J (2005). Fusion of similarity data in clustering. Advances in Neural Information Processing Systems 18.

[18] Chuang Y (2012). Affinity aggregation for spectral clustering. IEEE Conf Comput Vis Pattern Recogn.

[19] Gönen M, Margolin A (2014). Localized data fusion for kernel k-means clustering with application to cancer biology. Adv Neural Inf Process Syst.

[20] Bach F, Lanckriet G, Jordan M. Multiple kernel learning, conic duality, and the smo algorithm. In: International Conference: 2004. p. 6. 10.1145/1015330.1015424.

[21] Nigro JM, Misra A, Zhang L, Smirnov I, Colman H, Griffin C, Ozburn N, Chen M, Pan E, Koul D, Yung WKA, Feuerstein BG, Aldape KD (2005). Integrated array-comparative genomic hybridization and expression array profiles identify clinically relevant molecular subtypes of glioblastoma. Cancer Res.

[22] Verhaak Roel GW (2010). Integrated genomic analysis identifies clinically relevant subtypes of glioblastoma characterized by abnormalities in pdgfra, idh1, egfr, and nf1. Cancer Cell.

[23] Cai M, Li L. Subtype identification from heterogeneous tcga datasets on a genomic scale by multi-view clustering with enhanced consensus. BMC Med Genomics. 2017; 4:75. 10.1186/s12920-017-0306-x. 10.1186/s12920-017-0306-x PMC5763310 29322925

[24] Gretton A, Bousquet O, Smola A J, Schölkopf B. Measuring statistical dependence with hilbert-schmidt norms. In: ALT: 2005. p. 63–77. 10.1007/11564089_7.

[25] Bartels RH, Stewart GW (1972). Solution of the matrix equation ax+xb=c [f4] (algorithm 432). Commun Acm.

[26] Wen, Zaiwen (2013). A feasible method for optimization with orthogonality constraints. Math Program.

[27] Rousseeuw PJ (1999). Silhouettes: A graphical aid to the interpretation and validation of cluster analysis. J Comput Appl Math.

[28] Network CGA (2012). Comprehensive molecular characterization of human colon and rectal cancer. Nature.

[29] Network TCGA. The cancer genome atlas. 2006. http://cancergenome.nih.gov/. Accessed 20 Jun 2019.

[30] Parker JS, Mullins M, Cheang MC (2009). Supervised risk predictor of breast cancer based on intrinsic subtypes. J Clin Oncol.

[31] Curtis C, Shah SP, Chin SF, Turashvili G, Rueda OM, Dunning MJ, Speed D, Lynch AG, Samarajiwa S, Yuan Y (2012). The genomic and transcriptomic architecture of 2,000 breast tumours reveals novel subgroups. Nature.

[32] Pires MM, Hopkins BD, Saal LH, Parsons RE (2013). Alterations of egfr, p53 and pten that mimic changes found in basal-like breast cancer promote transformation of human mammary epithelial cells. Cancer Biol Ther.

[33] Holland DG, Burleigh A, Git A, Goldgraben MA, Perezmancera PA, Chin SF, Hurtado A, Bruna A, Ali HR, Greenwood W (2015). Znf703 is a common luminal b breast cancer oncogene that differentially regulates luminal and basal progenitors in human mammary epithelium. Embo Mol Med.

[34] Bentires-Alj M, Gil SG, Chan R, Wang ZC, Wang Y, Imanaka N, Harris LN, Richardson A, Neel BG, Gu H (2006). A role for the scaffolding adapter gab2 in breast cancer. Nat Med.

[35] Leehoeflich S, Pham T, Dowbenko D, Munroe X, Lee J, Li L, Zhou W, Haverty P, Pujara K, Stinson J (2011). Ppm1h is a p27 phosphatase implicated in trastuzumab resistance. Cancer Discov.

[36] Fisher R, Vol. 118. Statistical Methods for Research Workers; 1954, pp. 66–70. 10.2307/2528855.

